# Taxonomic and Ecological Interaction of Leishmaniasis Vectors (Diptera: Psychodidae) in Sefrou Province (Middle Atlas Morocco)

**DOI:** 10.1155/2022/9382154

**Published:** 2022-09-12

**Authors:** Fatima Zahra Talbi, Abdelkarim Taam, Hajar El Omari, Said Hilali, Mouhcine Fadil, Fatiha El Khayyat, Mohamed Najy, Meryem Mrani Alaoui, Fouad El-Akhal, Abdellatif Alami, Rachid Amaiach, Khadija Lahouiti, Amal Taroq, Abdelhakim El Ouali Lalami

**Affiliations:** ^1^Hassan First University of Settat, Faculty of Sciences and Technologies, Laboratory of Biochemistry, Neurosciences, Natural Resources and Environment, P.O. Box 577, Settat 26000, Morocco; ^2^Sidi Mohamed Ben Abdellah University, Faculty of Sciences Dhar El Mahraz, Laboratory of Biotechnology, Conservation and Valorization of Naturals Resources (LBCVNR), Fez 30000, Morocco; ^3^Laboratory of Engineering Sciences, National School of Applied Sciences (ENSA), Ibn Tofail University, Kenitra, Morocco; ^4^National Education, Preschool and Sports, Regional Academy of Education and Formation of Fez-Meknes, Qualifying Secondary Education, Moulay Ismail High School, Meknes, Morocco; ^5^Physio-Chemical Laboratory of Inorganic and Organique Materials (LPCMIO), Materials Science Center (MSC), Ecole Normale Supérieure, Mohammed V University in Rabat, Rabat, Morocco; ^6^Natural Resources and Sustainable Development Laboratory, Faculty of Sciences, Ibn Tofail University, BP133, Kenitra 14000, Morocco; ^7^Laboratory of Biochemistry, Environment and Agrifood, URAC36, Faculty of Sciences and Technologies, Hassan II University Casablanca, BP 146, Mohammedia 20650, Morocco; ^8^Institute of Nursing Professions and Health Techniques of Tetouan (Annex Al Hociema), Regional Health Directorate, Mohammed V Hospital, Al Hociema 32000, Morocco; ^9^Laboratory of Applied Organic Chemistry, Faculty of Science and Technology, Sidi Mohamed Ben Abdellah University, Fez, Morocco; ^10^Higher Institute of Nursing Professions and Health Techniques of Fez, Regional Health Directorate Fez-Meknes, El Ghassani Hospital, Fez 30000, Morocco; ^11^Laboratory of Microbial Biotechnology, Department of Biology, Faculty of Sciences and Technology, University Sidi Mohamed Ben Abdellah, BP 2202, Road of Immouzer, Fez, Morocco; ^12^Laboratory of Natural Substances, Pharmacology Environment, Modilesation, Health and Quality of Life, Faculty of Sciences Dhar Mahraz, University Sidi Mohamed Ben Abdellah, Fez, Morocco

## Abstract

An entomological survey was carried out in the locality of Aichoune to conduct a study on sand flies, species composition, and monthly relative abundance. This study is essential for the implementation of integrated vector management control. Insects collection was carried out twice a month from January 2013 to December 2014 by means of adhesive and CDC-type light traps. A total of 5441 sand flies were collected with the predominance of males (a sex ratio = 1.89). The sampled specimens consist of seven species divided into two genera: *Phlebotomus* (99.55%) and *Sergentomyia* (0.44%). *Phlebotomus sergenti* was the dominant species with an average annual proportion of 47.38%, followed by *P. perniciosus* (37.32%), *P. longicuspis* (8,56%), *P. papatasi* (6.23%), and *P. ariasi* (0.05%). The genus *Sergentomyia* was less common (0.44%). *S.minuta* was represented only by 0.36% and *S. fallax* by 0.07%. The species dynamics showed a unimodal evolution for *P. sergenti* and *P. papatasi*. They were active from May to October. *P. perniciosus* presents a trimodal trend showing the most relevant peak in August. The highest number of specimens was collected in June, when the temperature reaches an annual average value of 25.5°C. The obtained results will help us better understand the leishmaniasis transmission dynamics in the Aichoune locality and will contribute to the design of a surveillance strategy.

## 1. Introduction

Sand flies are Diptera Nematocera belonging to the family of Psychodidae, a subfamily of *Phlebotominae* [1]. Among approximately 900 species estimated less than a hundred, belonging to *Phlebotomus* and *Lutzomyia* genera are proven or suspected vectors of human disease in the Old and New Worlds, respectively [2–5]. Sand flies are widely spread, especially in Central America, South America, Africa, Asia, and Southern Europe. They cannot be found in New Zealand, the Pacific Islands, or the Nordic countries, and they are very rare in Australia and North America. The abundance of species varies according to latitude: throughout the year in the intertropical zone, but they appear only in summer in temperate regions, which gives the disease a seasonal trend [6, 7].

Among more than 50 *Phlebotomus* species described in Europe, North Africa, the Middle East, and the Caucasus, eleven are confirmed or suspected *Leishmania infantum* vectors [7].

In Morocco, sand flies and their geographical distribution have been studied by several authors since the beginning of the century. After the first partial analysis, Ristorcelli published four successive notes on the collection made by Langeron in Southern and Eastern Morocco [8]. In 1947, Gaud proved for the first time the presence and abundance of sand flies in Atlantic Morocco [9]. Additionally, in 1954, he developed a regional distribution and reporting seasonality and abundance of sand flies, based on the collection of 4,509 sand flies throughout the country. Parrot and Durand-Delacre carried out several studies on the biology of sand flies in the Western Saharian regions. Finally, the work of Bailly–Choumara et al. [10] developed a geographical and bioclimatic synthesis of sand flies in Morocco where three climatic parameters appear fundamental to explaining the biogeographical distribution of species: annual precipitation, average maximum temperature for the hottest month, and the average minimum temperature for the coldest month. *P. ariasi* is related to the humid stage [11] between 1000 and 1400 m, but no specimens were found below 1000 m in the South of the High Atlas [12]. *P. perniciosus* presence is related to mountain areas of humid, subhumid [13], and also in semiarid stages [8]. On the contrary, *P. papatasi* is present in the arid and Saharian stages [14]. *P. longicuspis* was collected at all altitudes, with a particularly high density recorded between 600 and 799 m. *P. sergenti* was abundant in the semiarid bioclimatic stage, as in the bordering areas of the arid region [14], and widespread between 800 and 900 m. Recently, the work of Guernaoui et al. [9, 15] and of Boussaa [16, 17] in southwestern Morocco made it possible to update Rioux's data and revise the distribution of the *Larroussisus* genus. In 2015, Kahime et al. [18] demonstrated the role of soil texture as a factor influencing sand flies' distribution. Sand flies have been rare and even absent in silty and clay-clay-silty soils, but they are very abundant in sandy-textured sites.

Several studies carried out in Morocco have shown the presence of 22 sand fly species; 13 belong to *Phlebotomus* and 9 to the *Sergentomyia* genus [19]. However, only five species threatening public health have been identified: *P. sergenti* L. *tropica* vector, *P. papatasis* L. *major* vector, *P. perniciosus*, *P. ariasi,* and *P. longicispus* L. *infantum* vectors [11, 20]. The female, which is hematophagous, can survive on a floricultural diet but needs a blood source for egg production [21]. Knowledge of the trophic preferences of female sand flies in the natural environment is essential for the evaluation of their vectorial capacity. The choice of the host depends on their availability, more specifically their number and size, rather than their specific attractiveness [22]. Some female sand flies are autogenous, such as *Phlebotomus papatasi* and *Lutzomyia gomezi* [23]. The aim of this work was to define sand flies' fauna distribution and to study their dynamics and phenology to better understand and control leishmaniasis diseases related to seasonal climatic factors.

## 2. Materials and Methods

### 2.1. Study Area

**Figure 1:**
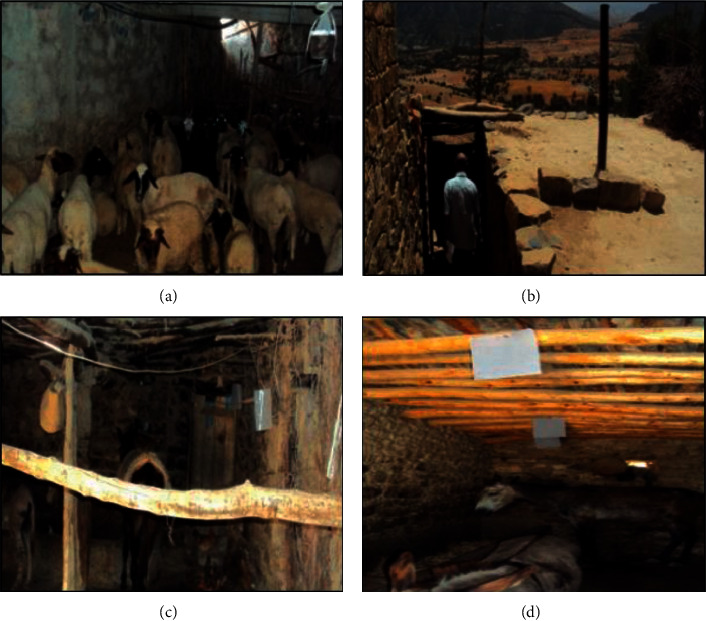
Different biotopes in the study area: (a) sheep pen, (b) the winery, (c) stable, (d) stable henhouse.

The mountain area investigated is located in the south of Sefrou Province in the Middle Atlas-Morocco, in Aichoun locality (33°39′N, 04°38′W). In order to determine the factors involved in the distribution and density of sand fly specimens and to have a representative sample of the whole area, traps were placed in different biotopes. Sites have been chosen based on epidemiological and ecological factors (Figure 1).

### 2.2. Kind and Design of Study

This study is an entomological survey. It was carried out bimonthly from January 2013 to December 2014. Different sampling methods were used for the collection [24]. Sheets of white paper (29.7 × 21 cm), coated with castor oil, were placed to catch resting females and CDC-light traps suitable for trapping active sand fly females from 18:00 PM to 06:00 AM. The traps were collected the following morning, and then the specimens were stored in 70% ethanol using mouth aspirators. The collected specimens were dissected and identified following morphological keys as suggested by the guide of the Ministry of Health, Lewis and Kahime et al. [18].

### 2.3. Data Analysis and Statistical Processing

Ecological (relative abundance and sex ratio) and meteorological parameters were used for quantitative and qualitative evaluation [25]. A Pearson's correlation test was performed by Statgraphics Centurion software (version XVI) to investigate the correlation between sand flies' abundance, average monthly temperature, and average monthly precipitation. A *p*value of less than 0.05 was considered to be statistically significant.

## 3. Results

### 3.1. Sand Fly Species Composition

**Figure 2:**
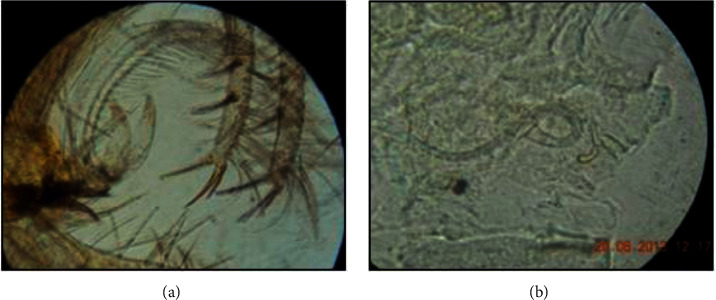
Details of *P. papatasi*: (a) male penile valves (*G* × 40); (b) female spermatheca (*G* × 100).

A total of 5441 specimens, which belong to seven species: five of the *Phlebotomus* genus and 2 of the *Sergentomyia* genus, were collected and identified. *P. sergenti* was the prevalent species with a prevalence of 47.38%, followed by *P. perniciosus* at 37.32%, *P. papatasi* (Figure 2) at 6.23%, and *P. longicuspis* at 8.56% (Table 1). Overall, the CDC light trap gave a higher percentage of 58.30% of specimens collected compared with sticky traps (41.70%). The male/female ratio was 1.89.

The total number of phlebotomine specimens caught in the Aichoune locality for each method is reported in Table 1. Prevalences of species caught with light traps were, *P. perniciosus* (30.08%)*, P. sergenti* (14.26%), *P. longicuspis* (7.35%), *P. papatasi* (3.17%), *P. ariasi* (0.05%), *S. minuta* (0.31%), and *S. fallax* (0.07%). Conversely, sticky tarps gave a higher prevalence of *P. sergenti* species (33.11%).

*P. perniciosus* species present a specific characteristic in males with a typical shape (bifurcated penile valves) and another so-called atypical form whose penile valves are slightly curved at their ends and not bifid. As a result, males of this species, having an atypical form, have been widely confused with the males of a sympatric species of *P. longicuspis*. The two forms of *P. perniciosus* are widespread in the Moroccan territory. In this respect, the typical morphotype of *P. perniciosus* is primarily distributed in the northen part of the country. However, the atypical morphology is found specifically in the South, where only 1 in 100 males has bifid penises [26].

### 3.2. Sand fly Dynamics

**Figure 3:**
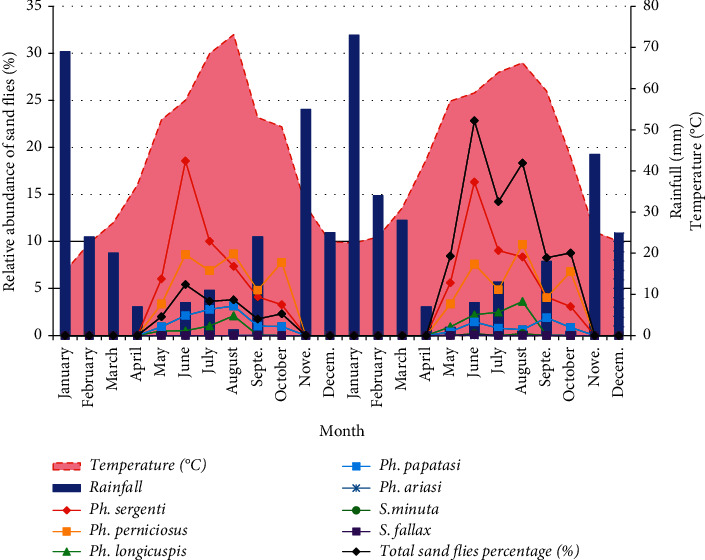
Seasonal dynamics of sand flies and the effect of the ecological parameters of Aichoune locality, Sefrou Province, Morocco.

The seasonal fluctuation of sand fly total catches for both surveyed years is represented by a trimodal distribution, showing three peaks in June, August, and October. In 2013, the three dominant species in the Aichoune locality, *P. sergenti*, *P. papatasi,* and *P. longicuspis*, exhibited a mono-modal activity distribution with a very remarkable peak during the month of June. Meanwhile, *P. perniciosus* showed a trimodal peak distribution in June (8.6%), August (8.68%), and October (7.78%). The other species were present in a low proportion (Figure 3).

Their presence was markedly important due to their vectorial capacity even if their proportion was reduced. *P. papatasi* followed a heterogeneous seasonal evolution, mono-modal in 2013 showing a maximum abundance in the month of August.

During 2014, the period of seasonal activity of sand flies was not changed; it is spread over six months, from May to October. But the fluctuation in seasonality has changed for certain species. The two predominant species in the study area *P. sergenti* and *P. longicuspis* displayed a mono-modal activity distribution with a remarkable peak during the same months of June (16.32%) and August (3.62%). *P. perniciosus* still showed a trimodal peak distribution in June (7.6%), August (9.68%), and October (6.78%). The other species were always present but in a low proportion. On the other hand, *P. papatasi* modified its mode of evolution (bimodal) and traced two peaks respectively in June (1.45%) and September (1.9%) (Figure 3).

### 3.3. Influence of Ecological Parameters on Sand Flies' Dynamics

In our study area, the activity of sand fly fauna appears only from May until October, which corresponds with the hot season. In general, the majority of the collected species were present with an important relative abundance during June and August, when the average temperature of the two years was respectively 25.4°C and 30.5°C (Figure 3). From the meteorological data, we were able to reveal the coexistence of two seasons. A dry period lasts five months from November to March, and a cold period of seven months from April to October. August was considered to be the driest month, while January was recorded as the wettest. According to the obtained results, precipitation and temperature could be the limiting parameters during the cold period. Indeed, no sand fly species are present when the rate reaches an average value of two years, which is between 24 mm/6.84°C and 73 mm/18.71°C (Figure 3).

The purpose of this section was to study the statistical correlations between the relative abundances of the species and their correlations with climatic factors, i.e., temperature and rainfall. A positive correlation between the relative abundance of two species testifies to their coexistence in the same area under the same conditions, whereas a significant correlation between relative abundance and a climatic factor indicates a significant influence of this parameter. Thus, the results show that the majority of the species were statistically correlated between them, except for *P. ariasi*, which was correlated with *S. fallax* only (*r* = 0,46; *p*-value <0,05). Our findings show that temperature has a significant positive correlation with the relative abundance of the species *P. sergenti* (*r* = 0,75; *p* value <0,001), *P. perniciosus* (*r* = 0,86; *p*-value <0,001), *P. longicuspis* (*r* = 0,69; *p* value <0,001) and *P. papatasi* (*r* = 0,81; *p* value <0,001). While rainfall has a significant negative correlation with the relative abundance of the species *P. sergenti* (*r* = −0,49; *p* value = 0,01), *P. perniciosus* (*r* = −0,62; *p*-value = 0,001), *P. longicuspis* (*r* = −0,41; *p* value = 0,04) and *P. papatasi* (*r* = −0,4739; *p* value = 0,01).

These outcomes indicate that the numbers of these four species increase significantly with temperature and decrease significantly with rainfall. In other words, the temperature and rainfall can be considered as favoring factors and limiting factors, respectively. Otherwise, the three species (*P. ariasi*, *S. minuta,* and *S. fallax*) seem not to be influenced by these two climatic parameters.

## 4. Discussion

*P. sergenti*, *P. papatasi*, *P. longicuspis*, *P. perniciosus,* and *P. ariasi*, are species potentially implicated in the transmission of different leishmaniasis etiological entities in Morocco [8]. These species are also incriminated in Tunisia and eastern Saudi Arabia [27].

In the Aichoune locality, seasonal fluctuation of sand flies showed the richness of species during 2013 and 2014, with a bimodal distribution peak in June and July for total sand flies trapped. In 2013, the three dominant species, *P. sergenti*, *P. papatasi,* and *P. longicuspis*, exhibited a mono-modal activity. Meanwhile, *P. perniciosus* showed a trimodal trend. The other species were present in a low proportion. The same result was obtained in 2014 except for *P. papatasi* which showed bimodal activity.

Generally, in Morocco, *P. papatasi* is relayed to the south by two closely related species, *P. bergeroti* and *P. duboscqi*. However, it has been found to be relatively very common in our collection. This species is more adapted to the arid and Saharan environments [16, 28]. *P. papatasi* is widely distributed in areas where the temperature varies between 16°C and 44°C and can be collected at altitudes ranging from sea level up to over 1500 m [14].

*P. longicuspis* is a sand fly well adapted to Mediterranean conditions. It is characterized by a wide geographical distribution thanks to its ecological plasticity. It is also suspected as *L. infantum* vector in Mediterranean countries [13]. Its vectors' role has been proven in Algeria [29] and Morocco [30]. In Morocco, this species is collected at various bioclimatic levels and at all altitudes. It shows good adaptation to climatic changing conditions in the Mediterranean basin (Algeria, Libya, Morocco, Spain, Tunisia). In the study area, the highest density of *P. longicuspis* was observed in August in both surveyed years. This result is in accordance with Guernaoui [15] in Chichaoua province, where this species showed a mono-modal distribution, with one density peak in August-September. The statistical study in this work shows that this species is statistically correlated with the other medically interesting species collected from the *Phlebotomus* genus: *P. sergenti* (*r* = 0,63; *p*value = 0,0008); *P. perniciosus* (*r* = 0,66; *p*value = 0,0003) and *P. papatasi* (*r* = 0,42; *p*value = 0,03) except for *P. ariasi* species (*r* = 0,1; *p*value = 0,61).

In our study, *P. perniciosus* species were recorded with a high relative abundance (37.32%). *P. ariasi* is an uncommon species that is present in a humid and subhumid climate, especially in mountainous regions [8]. It has been proven as a vector of leishmaniasis in northern Morocco in the Province of Taounate [31]. This species was collected at a very low density in the Aichoune region (0.05%).

The other species found belong to the *Sergentomyia* genus. They are zoophilic species that bite humans only occasionally. For this reason, they do not play any epidemiological role. Females feed primarily on reptiles, birds, and amphibians [12]. For this reason, they are involved in the transmission of sauro leishmaniases to reptiles. The species of the *Sergentomyia* genus, *S. minuta* (0.36%) and *S. fallax* (0.07%), were collected at a very low density during our captures in the Aichoune region. In Morocco, this species is widespread [32]. It was collected at a very low density at the Aichoune locality [26]. In 2012, in the same locality, the distribution of *P. sergenti*, *P. perniciosus,* and *P. papatasi*, followed by a biphasic mode, with the first peak in July and the second in September [12]. Indeed, *P. sergenti* is very abundant throughout the territory of Morocco [14], whereas its wide distribution is localized in arid and subhumid climates [33]. In Aichoune, *P. sergenti* followed a mono-modal seasonal evolution with a single remarkable peak in June (18.56% in 2013 and 16.32% in 2014). The absence of a second peak could be due to the effect of ecological factors. According to Cross et al. [34] and Ghosh et al. [35], the seasonal distribution is favored by local environmental factors (precipitation and temperature), biotic factors (abundance and dynamics of vertebrate hosts), and physical factors (habitat availability). Climatic factors would play a very important role in sand fly dynamics observed in species caught during the study period. According to climatic data, in the Aichoune locality, precipitation could be a limiting parameter during the cold period. No sand fly species are present when the rate reaches an average value between 24 mm and 71 mm. Nevertheless, all species were present with a high relative abundance especially in June and August when the temperature reaches 25.41°C and 30.5°C. Thus, a positive significant correlation has been detected between temperature and vector species present in the study area.

In the region of Fes-Boulemane, particularly in Moulay Yaacoub Province in Oulad Aid, temperature and humidity are confirmed to be the main factors behind the spread of the disease [36].

## 5. Conclusions

The presented results have contributed in updating sand fly species compositions of Central Morocco and particularly sand fly potential vectors of leishmaniasis in the locality of Aichoune. The study allowed us to identify the environmental factors influencing the species proliferation. Thus, we observed the existence of 7 species, which represent 30.43% of the total sand fly population of Morocco. This fauna belongs to two genera: *Phlebotomus* and *Sergentomyia*. The first genus was represented by five species: *P. perniciosus*, *P. sergenti*, *P. longicuspis*, *P. papatasi*, and *P. ariasi*. The second is with only two species: *S. minuta* and *S. fallax*. This study confirms Leishmanian risk in this area and suggests the implementation of an integrated and specific strategy to control the spread of sand fly vectors in the Aichoune locality.

## Figures and Tables

**Table 1:** Sand fly species composition in Aichoune.

Subgenus	Species	Sticky traps collection			Total	CDC light traps collection			Total
		*M*	*F*	Ratio sex		*M*	*F*	Ratio sex	
*Larroussius*	*P. longicuspis*	17	49	0.34	66	345	55	6.27	400
	*P. perniciosus*	180	51	3.52	231	945	855	1.10	1800
	*P. ariasi*	0	0	0	0	1	2	0.50	3

*Paraphlebotomus*	*P. sergenti*	1486	316	4.70	1802	386	390	0.98	776

*Phlebotomus*	*P. papatasi*	115	51	2.25	166	76	97	0.78	173

*Sergentomyia*	*S. minuta*	0	3	0	3	11	6	1.83	17
	*S. fallax*	0	0	0	0	0	4	0	4

## Data Availability

The data used to support the findings of this study are included within the article.

## References

[1] Benallal K. E., Garni R., Harrat Z., Volf P., Dvorak V. (2022). Phlebotomine sand flies (Diptera: Psychodidae) of the Maghreb region: a systematic review of distribution, morphology, and role in the transmission of the pathogens. *PLoS Neglected Tropical Diseases*.

[2] Ready P. D. (2013). Biology of phlebotomine sand flies as vectors of disease agents. *Annual Review of Entomology*.

[3] Young D. G., Duncan M. A. (1994). Guide to the identification and geographic distribution of Lutzomyia sand fliesin Mexico, the West indies, central and South America (Diptera: Psychodidae). *Memoirs of the American Entomological Institute*.

[4] Gradoni L. (2017). The leishmaniases of the Mediterranean region. *Current Tropical Medicine Reports*.

[5] Berriatua E., Maia C., Conceição C. (2021). Leishmaniases in the European union and neighboring countries. *Emerging Infectious Diseases*.

[6] Dedet P. (2001). Les leishmanioses. *Encyclopédie médico-chirurgicale, Maladies infectieuses, 8094 A10*.

[7] Alten B., Maia C., Afonso M. O. (2016). Seasonal dynamics of phlebotomine sand fly species proven vectors of mediterranean leishmaniasis caused by leishmania infantum. *PLoS Neglected Tropical Diseases*.

[8] Faraj C., Adlaoui E. B., Ouahabi S. (2013). Distribution and bionomic of sand flies in five ecologically different cutaneous leishmaniasis foci in Morocco. *ISRN Epidemiology*.

[9] Guernaoui S., Pesson B., Boumezzough A., Pichon G. (2005). Distribution of phlebotomine sandflies, of the subgenus Larroussius, in Morocco. *Medical and Veterinary Entomology*.

[10] Bailly-Choumara H., Abonnenc E., Pastre J. (1971). Contribution á l’étude des phlébotomes du Maroc. (Diptera: Psychodidae): données faunistiques et écologiques. *Cah ORSTOM. Ser Ent Med Parasitol*.

[11] Talbi F. Z., El Ouali Lalami A., Fadil M. (2020). Entomological investigations, seasonal fluctuations and impact of bioclimate factors of phlebotomines sand flies (Diptera: Psychodidae) of an emerging focus of cutaneous leishmaniasis in Aichoun, central Morocco. *Journal of Parasitology Research*.

[12] Talbi F. Z., El Ouali Lalami A., Janati Idrissi A., Sebti F., Faraj C. (2015). Leishmaniasis in central Morocco: seasonal fluctuations of phlebotomine sand fly in Aichoun locality, from Sefrou province. *Pathology Research International*.

[13] Berchi S. (1990). *Ecologie des phlébotomes (Diptera, Psychodidae) de l’Est algérien, mémoire de Magister en Entomologie Appliquée*.

[14] Killick-Kendrick R. (1990). Phlebotomine vectors of the leishmaniases: a review. *Medical and Veterinary Entomology*.

[15] Guernaoui S. (2006). Les leishmanioses dans les zones arides et semi-arides du Sud-ouest marocain. *Ecologie, Épidémiologie, Modélisation et Aide à la Décision Pour la Lutte Antivectorielle*.

[16] Boussaa S. (2008). *Epidémiologie des Leishmanioses Dans la Région de Marrakech, Maroc: Effet de L’urbanisation sur la Répartition Spatio-Temporelle des Phlébotomes et Caractérisation Moléculaire de Leurs Populations*.

[17] Boussaa S., Guernaoui S., Boumezzough A. Periodicity pf phlebotomine sand flies in an urban area of Marrakech, Morocco.

[18] Kahime K., Boussaa S., El Mzabi A., Boumezzough A. (2015). Spatial relations among environmental factors and phlebotomine sand fly populations (Diptera: Psychodidae) in central and southern Morocco. *Journal of Vector Ecology*.

[19] Anonyme (2010). *Lutte contre les leishmanioses, Guide des activités, Direction de l’épidémiologie et de lutte contre les maladies, Service des Maladies parasitaires*.

[20] Maroli M., Feliciangeli M. D., Bichaud L., Charrel R. N., Gradoni L. (2013 Jun). Phlebotomine sand flies and the spreading of leishmaniases and other diseases of public health concern. *Medical and Veterinary Entomology*.

[21] Killick-Kendrick R., Killick-Kendrick M. (1999). *Biology of Sand Fly Vectors of Mediterranean Canine Leishmaniasis*.

[22] Bongiorno G., Habluetzel A., Khoury C., Maroli M. (2003). Host preferences of phlebotomine sand flies at a hypoendemic focus of canine leishmaniasis in central Italy. *Acta Tropica*.

[23] Schlein Y., Jacobson R. L. (1999). Sugar meals and longevity of the sand fly Phlebotomus papatasi in an arid focus of Leishmania major in the Jordan valley. *Medical and Veterinary Entomology*.

[24] Alexander B. (2000). Sampling methods for phlebotomine Sand flies. *Medical and Veterinary Entomology*.

[25] Artemiev M. M. (1991). A classification of the subfamily Phlebotominae. *Parassitologia*.

[26] Talbi F. Z., Faraj C., El-Akhal F. (2015). Diversity and dynamics of sand flies (Diptera: Psychodidae) of two cutaneous leishmaniasis foci in the fes-boulemane region of northern Morocco. *International Journal of Zoology*.

[27] Jose A. (2010). Ruiz postigo, leishmaniasis in the world health organization eastern Mediterranean region. *International Journal of Antimicrobial Agents*.

[28] Rioux J. A. (2001). Trente ans de coopération franco-marocaine sur les leishmanioses: Dépistage et analyse des foyers. Facteurs de risque. Changements climatiques et dynamique noso-géographique. *Revue de l’Association des Anciens Élèves de l’Institut Pasteur*.

[29] Es-Sette N., Ajaoud M., Laamrani-Idrissi A., Mellouki F., Lemrani M. (2014). Molecular detection and identification of leishmania infection in naturally infected sand flies in a focus of cutaneous Leishmaniasis in northern Morocco. *Parasites & Vectors*.

[30] Hamdani A. (1999). *Etude de la faune phlébotomienne dans trois foyers de leishmanioses au Nord du Maroc: Espèces, Abondance, Saisonnalité et incrimination du vecteur, Thèse de 3ème cycle*.

[31] Boussaa S., Kasbari M., El Mzabi A., Boumezzough A. (2014). Epidemiological investigation of canine leishmaniasis in southern Morocco. *Advances in Epidemiology*.

[32] Daoudi M. M., Boussaa S., Boumezzough A. (2020). Modeling spatial distribution of sergentomyia minuta (Diptera: Psychodidae) and its potential implication in leishmaniasis transmission in Morocco. *Journal of Arthropod-Borne Diseases*.

[33] Rioux J. A. (1999). Eco-epidemiology of leishmaniasis in Morocco: review of 30 years of cooperation. *Epidemiological Bulletin*.

[34] Cross E. R., Newcomb W. W., Tucker C. J. (1996). Use of weather data and remote sensing to predict the geographic and seasonal distribution of Phlebotomus papatasi in southwest Asia. *The American Journal of Tropical Medicine and Hygiene*.

[35] Ghosh K. N., Mukhopadhyay J. M., Guzman H., Tesh R. B., Munstermann L. E. (1999). Interspecific hybridization and genetic variability of *Phlebotomus* sandflies. *Medical and Veterinary Entomology*.

[36] Lahouiti K., El Ouali Lalami A., Maniar S., Bekhti K. (2013). Seasonal fluctuations of phlebotomines sand fly populations (Diptera: Psychodidae) in the Moulay Yacoub Province, centre Morocco: effect of ecological factors. *African Journal of Environmental Science and Technology*.

